# Retraction Note: Gamma-radiated immunosuppressed tumor xenograft mice can be a new ideal model in cancer research

**DOI:** 10.1038/s41598-023-46583-1

**Published:** 2023-11-15

**Authors:** Hamid Khodayari, Saeed Khodayari, Solmaz Khalighfard, Arash Tahmasebifar, Mahboubeh Tajaldini, Amirhoushang Poorkhani, Hassan Nikoueinejad, Gholam Ali Hamidi, Hassan Nosrati, Mohammad Reza Kalhori, Ali Mohammad Alizadeh

**Affiliations:** 1 Physiology Research Center, Kashan University of Medical Sciences, Kashan, Iran; 2 Radiation Oncology Research Center, Tehran University of Medical Sciences, Tehran, Iran; 3 Cancer Research Center, Tehran University of Medical Sciences, Tehran, Iran; 4 Ischemic Disorder Center, Golestan University of Medical Sciences, Gorgan, Iran; 5 Nephrology and Urology Research Center, Baqiyatallah University of Medical Sciences, Tehran, Iran; 6 Medical Biology Research Center, Kermanshah University of Medical Sciences, Kermanshah, Iran; 7 Breast Disease Research Center, Tehran University of Medical Sciences, Tehran, Iran

Retraction of: *Scientific Reports* 10.1038/s41598-020-80428-5, published online 08 January 2021

The Editors have retracted this Article. Concerns were raised regarding a number of figures, specifically:

- Figure 1c: panel b appears to overlap with the 29 day/CNTs panel in Figure 10 of^1^ and the 28 Day OT panel in Figure 1 of^2^.
- Figure 1c: panel c appears to overlap with the 14 day/control panel and the 21 day/ATO panel of Figure 1D in^2^.

Further checks by the Publisher have found that the error bars in Figures 1, 3 and 4 are ± 5%, not SD as stated in the Materials and Methods and Figure legends.

The Editors therefore no longer have confidence in the results and conclusions of this Article.

Ali Mohammad Alizadeh does not agree to this retraction. None of the other authors have responded to any correspondence from the Publisher about this retraction.
